# Polysplenia syndrome revealed in adulthood by pancreatic and vascular malformations: a case report

**DOI:** 10.11604/pamj.2022.43.77.31496

**Published:** 2022-10-12

**Authors:** Meryem El Mountassir, Mohamed Borahma, Imane Benelbarhdadi, Nawal Lagdali, Abdelmoughit Hosni, Fatima Zahra Ajana

**Affiliations:** 1 Mohammed V University, Ibn Sina Hospital, Department of Gastroenterology C, Rabat, Morocco,; 2 Mohammed V University, Ibn Sina Hospital, Department of Emergency Radiology, Rabat, Morocco

**Keywords:** Imaging, multiple spleens, polysplenia syndrome, case report

## Abstract

Polysplenia syndrome (PSS) is a rare congenital disease that associates multiple spleens to other malformations, most frequently cardiac, vascular, visceral, and biliary malformations. Most patients with PSS die in the early neonatal period because the disease is often accompanied by serve cardiac and biliary abnormalities. However, some patients have only mild cardiovascular malformations or anomalies in the abdominal organs, which are typically diagnosed incidentally in adulthood. We report the case of a 54-year-old woman who consulted for chronic atypical diffuse abdominal pain. The clinical examination was normal. Abdominal computed tomography showed a total of 5 spleens with vascular and pancreatic malformations as part of polysplenia syndrome. Symptomatic treatment was instituted with good evolution. No specific therapeutic indication was indicated in our case discovered incidentally in adulthood.

## Introduction

Polysplenia syndrome is a rare congenital disorder, often discovered incidentally in adults during imaging workup. It is characterized by the presence of multiple spleens associated with a variable spectrum of abdominal organ rotation abnormalities and cardiovascular and pulmonary system abnormalities [1]. Technical advances in sonography, computed tomography have greatly enhanced our ability to detect and characterize these anomalies [2]. We report through this case the essential place of computed tomography (CT) scan in the diagnosis and the lesion assessment of the whole abnormalities of this rare syndrome revealed in a woman in adulthood.

## Patient and observation

**Patient information:** a 54-year-old female patient with a history of type 2 diabetes and high blood pressure under treatment for 5 years, hypothyroidism on levothyrox for 15 years, no specific surgical or family history.

**Clinical findings:** she was admitted to our gastro-enterology department with a history of vaguely located chronic abdominal pain. The abdominal examination was normal.

**Figure 1:**
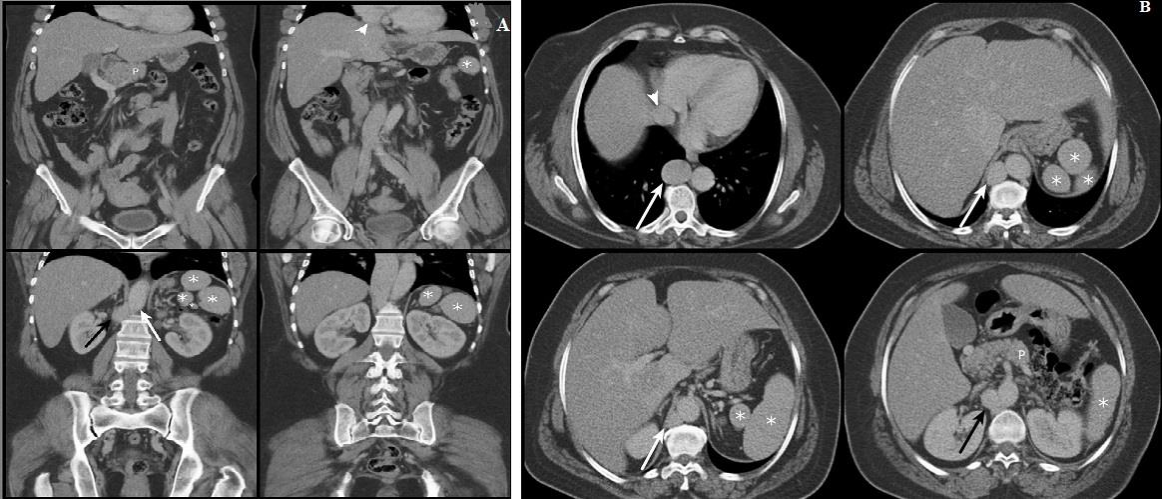
computed tomography scan with coronal (A) and axial (B) views showing the different anomalies found in the polysplenia syndrome in our patient

**Diagnostic assessment:** ultrasonography was first performed, revealing multiple echogenic masses in the left hypochondrium which may correspond to multiple spleens. Then, computed tomography was performed showing a total of 5 spleens located in the left hypochondrium (asterisks, Figure 1). Moreover, there was agenesis of the inferior suprarenal vena cava (black arrow, Figure 1) with a dilated azygos continuation (white arrow, Figure 1). The hepatic veins were flowing directly into the right atrium (arrowhead, Figure 1). The portal trunk was of normal caliber. The pancreas was short, with agenesis of its caudal segment (P on Figure 1), showing a pancake appearance. All these malformations are united in a single syndrome which is rare: polysplenia syndrome.

**Therapeutic interventions and follow-up:** symptomatic treatment was instituted for her abdominal pain with a good evolution, and no therapeutic management was indicated in this patient as these malformations were discovered incidentally in adulthood.

**Patient consent:** informed consent was obtained for this case report.

## Discussion

Polysplenia syndrome (PSS) is a rare congenital disease initially described by Helwig in 1929 [2,3]. Since then, few cases were described in the literature with an incidence of 1/250,000 live births [4]. The exact cause of polysplenia has not been clearly defined. Embryonic, genetic, and teratogenic components have all been implicated as causative factors in polysplenia [5]. Inferior vena cava (IVC) interruption with azygous continuation is the second most common abnormality observed in polysplenia patients, after multiple spleens [6]. Our patient had 05 spleens with agenesis of the inferior suprarenal vena cava with dilated azygos continuation. To understand this anomaly, it should be noted that the embryologic development of IVC is complex, and the normal IVC is composed of 4 segments: hepatic, suprarenal, renal, and infrarenal. The hepatic segment develops from the vitelline vein. The suprarenal segment is formed by the right subcardinal vein and subcardinal-hepatic anastomosis. The renal segment develops from the right supra-subcardinal and postsubcardinal anastomoses. The infrarenal segment derives from the right supracardinal vein [7]. Interruption of the IVC results from failure of the right subcardinal-hepatic anastomosis with consequent atrophy of the subcardinal vein (suprarenal IVC) and continuation of the infrarenal IVC as the azygous vein [7]. The suprahepatic segment of the IVC is usually present and drains separately into the right atrium [8] which is the case with our patient who had the hepatic veins flowing directly into the right atrium.

On the other hand, anomalies of the pancreas have been described in polysplenia syndrome. Normal pancreas formation occurs from fusion of ventral and dorsal pancreatic buds. The ventral pancreatic bud gives rise to the uncinate process and the head, while the dorsal pancreatic bud gives rise to the body and tail. The development of both dorsal pancreatic bud and spleen occur in the dorsal mesogastrium. Consequently, anomalies in both these organs can be expected in patients with polysplenia syndrome [9]. Most case series report a high incidence of short or truncated pancreas, which is the case of our patient, where only the pancreatic head with or without a small portion of the pancreatic body is present. The clinical relevance includes an increased incidence of pancreatitis and diabetes mellitus [6]. There is no clinical picture characteristic of PSS. There are no specific biological signs, nor hypersplenism linked to the increased splenic volume. The most frequently found clinical signs are generally related to associated biliopancreatic abnormalities (ulcers due to bile reflux, pancreatitis). There was no specific therapeutic indication for our patient, notably surgical, apart from symptomatic treatment of his pain with a good evolution, but during surgery, we should know that the malformative polymorphism requires the search for associated malformations and a rigorous control of the anatomy in order to avoid any intraoperative accident. A morphological assessment by thoraco-abdomino-pelvic CT scan with thoracic and abdominal CT angiography more or less coupled with MRI angiography is essential preoperatively [10].

## Conclusion

Diagnosis of polysplenia syndrome in adulthood is rare. The most common abnormalities are multiple spleens and IVC interruption with azygous continuation. We must know how to evoke it despite its rarity and request radiological examinations which are useful for diagnosis by determining the location and number of spleens, the location of other organs in the chest and abdomen and the identification of other associated abnormalities, especially preoperatively if there is a surgical indication.

## References

[1] Ghadouani F, Kharasse G, El Harroudi T, Sbai A, Talby K (2013). Syndrome de polysplénie avec association exceptionnelle polysplénie-diastématomyélie. Feuillets de Radiologie.

[2] Applegate KE, Goske MJ, Pierce G, Murphy D (1999). Situs revisited: imaging of the heterotaxy syndrome. Radiographics.

[3] Chen S-J, Li JW, Wang JK, Wu MH, Chiu IS, Chang CI (1998). Usefulness of electron beam computed tomography in children with heterotaxy syndrome. The American journal of cardiology.

[4] Rasool F, Mirza B (2011). Polysplenia syndrome associated with situs inversus abdominus and type I jejunal atresia. APSP journal of case reports.

[5] Durmaz M, Cengiz A, Arslan S, Erdogan H, Tolu I, Cengiz A (2016). An incidental findings of polysplenia syndrome in an adult patient with multiple anomalies. Clin Med Rev Case Rep.

[6] Fulcher AS, Turner MA (2002). Abdominal manifestations of situs anomalies in adults. Radiographics.

[7] Kandpal H, Sharma R, Gamangatti S, Srivastava DN, Vashist S (2008). Imaging the inferior vena cava: a road less traveled. Radiographics.

[8] Bass JE, Redwine MD, Kramer LA, Huynh PT, Harris Jr JH (2000). Spectrum of Congenital Anomalies of the Inferior Vena Cava: Cross-sectional Imaging Findings 1: (CME available in print version and on RSNA Link). Radiographics.

[9] Kapa S, Gleeson FC, Vege SS (2007). Dorsal pancreas agenesis and polysplenia/heterotaxy syndrome: a novel association with aortic coarctation and a review of the literature. JOP.

[10] Yilmaz E, Gulcu A, Sal S, Obuz F (2003). Interruption of the inferior vena cava with azygos/hemiazygos continuation accompanied by distinct renal vein anomalies: MRA and CT assessment. Abdominal imaging.

